# The ecology of medical care in Shanghai

**DOI:** 10.1186/s12913-020-06022-7

**Published:** 2021-01-09

**Authors:** Xuechen Xiong, Xiaolin Cao, Li Luo

**Affiliations:** Collaborative Innovation Center of Health Risks Governance, School of Public Health, Fudan University, Shanghai, 200433 China

**Keywords:** Ecology, Medical care, Healthcare seeking behavior, Shanghai

## Abstract

**Background:**

To better understand the distribution and consumption patterns of resources in different ethnic groups and at different levels of economic development, this paper chose to describe the healthcare seeking behavior in Shanghai.

**Methods:**

The data are from the Sixth Health Service Survey of Shanghai, which encompasses 23,198 permanent residents. Descriptive analyses were conducted to estimate the number of patients who reported health-related symptoms and healthcare-seeking behaviors per 1,000 residents. Logistic regression analyses were conducted to examine differences in reporting health-related symptoms and healthcare-seeking behaviors by age, gender and area of residence.

**Results:**

This paper have mapped the ecology of healthcare in Shanghai in 2018. Of 1000 individuals considered during a 1-month period, 444 reported sickness, 433 received treatment, 288 went to medical institutions, 195 went to primary medical institutions, 86 took a self-healing approach, 26 received TCM services, 7 were hospitalized, and 3 underwent surgery.

**Conclusions:**

Age is a risk factor leading to disease, medical treatment, self-medication, medical institution visits, TCM service, hospitalization and surgery. But age is a protective factor in the use of primary health care services. By gender, the number of people receiving medical services was similar, but women were statistically more likely to have surgery. As the income level increased, the number of patients and people receiving medical services showed a decreasing trend. Compared with the local population, the probability of non-local people visiting medical institutions was lower and statistically significant. Compared with the people who had health insurance, fewer uninsured people reported sickness and utilized healthcare services.

## Background

The theory of “the ecology of medical care” was first proposed by White in 1961 [1], providing a framework for understanding patterns of health-related symptoms experienced in specific populations of interest, as well as individual’s choices in seeking medical care, [2] in which the number of people among the total population who had utilized medical services in a given period of time is calculated. During the past decades, the theory of “the ecology of medical care” is popular in academic circles worldwide. Many countries and regions, such as Austria [3], Japan [4], USA [5], Hong Kong [6], and Beijing [7], have conducted surveys and applied this theory to describe local ecology of medical care. Furthermore, the analysis framework has been extensively applied to medical services for a certain group of patients, such as pediatric care [8], asthma patient care [9], and mental health care [10].

The past decade has seen a new round of medical reform in China, during which many new policies have emerged that may affect people’s medical behavior [11–13]. As the economic center of China, Shanghai has made remarkable achievements in China’s medical reform process in the past decade [14]. Several reports assessed the progress of Shanghai’s medical care systems, in the wake of rapid economic growth, significantly increased life expectancy, massively decreased infant mortality rate, greatly improved accessibility of medical resources [15]. However some reports also indicated that there are several undesirable features in Shanghai’s health outcome, such as waste of medical resources, inequities in access to health care [16], different health status across regions of different socioeconomic standings [17]. However, there is no research describing the framework of utilization of medical resources in Shanghai. This conceptual framework can help understanding ecological relationship and guide assessments of the adequacy, efficiency and appropriateness of the existing health care resources [18, 19].

In the previous studies of China’s health ecosystem, there was a study on Beijing’s health ecosystem [7]. In this research, on average per 1,000 adults, 295 had at least one symptom, 217 considered seeking medical care, 173 consulted a physician, 127 visited a hospital-based outpatient clinic, 43 visited a primary care physician, and 15 were hospitalized. However, there is no research on the health ecosystem in other cities of the Chinese mainland. However, there is no research on the health ecosystem in Shanghai. To better understand the distribution and consumption patterns of resources in different ethnic groups and at different levels of economic development, this paper chose to describe the healthcare seeking behavior and utilization of health services in Shanghai in comparison with other countries.

## Methods

### Data acquisition and study population

The data of Shanghai residents in this study are from the Sixth Health Service Survey of Shanghai in 2018. The National Health Service Survey began in 1993 and is conducted every five years. The main purpose of the National Health Service Survey was to evaluate the performance of health reform and development in the last five years. Thus, the National Health Service Survey will provide basic information for the implementation and evaluation of the new round of deepening reform of the medical and health system and provide the basis for the formulation of medium- and long-term plans and major action plans for health undertakings.

Multistage stratified cluster random sampling was used in the health service survey of Shanghai. According to this sampling principle, all 16 districts of Shanghai were participated in the survey. 5 streets will be drawn from each district, 2 neighborhood committees will be drawn from each street, and 60 households will be drawn from each neighborhood committee. A total of 600 households will be drawn from each district. Considering that the Pudong New District accounts for a high proportion of the population in Shanghai, in actual practice 1,200 households will be selected from the Pudong New District. Finally, A total of 23,198 permanent residents in the survey households were interviewed one-on-one by trained and qualified investigators. The on-site investigation work was mainly undertaken by the medical staff of the community health service centers, with investigators and investigation instructors. The investigator was responsible for the household survey, and the survey instructor was responsible for the organization, guidance, inspection and acceptance of the survey.

This survey was retrospective: the questions about illness and outpatient treatments were asked in reference to the previous 2 weeks. Hospitalization and surgery information was obtained from institutions for the previous year.

### Variable definitions

Based on the ecology model proposed by White, this paper estimated the proportion of the population in Shanghai who utilized medical services in 2018. The analysis was extended to different settings. To describe the ecology of healthcare, this paper used variables computed from the Sixth Health Service Survey of Shanghai in 2018 and estimated the number of residents per 1000 who, in an average month, received healthcare in each of 8 types of settings: (1) reported illnesses; (2) received treatment; (3) visited a physician in a medical institution (4) visited a physician in a primary medical institution; (5) took a self-healing approach; (6) received Traditional Chinese Medicine (TCM) treatment [20, 21]; (7) had a hospital stay; or (8) had surgery. There are four conditions to judge whether a respondent report illnesses. First, the respondent went to a medical institution for treatment due to illness or injury; Second, he/she consulted a doctor through the Internet because of discomfort; Third, he/she took self-treatment for the illness or injury; Fourth, he/she was suspended from work, school or bed rest for one day or more due to physical discomfort. One of the above four conditions is considered to report illness. To receive treatment means to go to a medical institution for medical treatment or self-treatment due to illness or injury, such as medication or physiotherapy. According to the level of medical institutions visited, medical institutions can be classified into primary medical institutions, regional medical institutions and tertiary medical institutions. Thus, a visit to physician in a primary medical institution refers to a patient’s choice of a primary health care facility for institutional treatment. According to the types of services, among the patients receiving medical services, if there are any items served by TCM in the treatment services, the patients will be classified as having received TCM services. As a whole, we can divide healthy ecosystems into three categories. The first category is the subjective health feedback of the population, that is, the indicators of reported illness. The second category is the use of outpatient services, mainly including self-treatment, grass-roots treatment, hospital treatment, traditional Chinese medicine treatment and so on. The third category is the utilization of inpatient services, mainly including hospitalization and surgery.

Predictor variables in multivariable analyses were taken from the Sixth Health Service Survey of Shanghai in 2018. This paper evaluated the following for inclusion as independent variables in multivariable analyses. (1) Age. There were 5 age groups: 0–17 years, 18–39 years, 40–64 years, 65–79 years and over 80 years. (2) Gender. (3) Economic status. Family income was divided into 5 categories: annual household income < 100,000 RMB, 100,000–300,000 RMB, 300,000–500,000 RMB, > 500,000 RMB). (4) Census register. There were two groups: One group consisted of registered residents of the population of Shanghai, and the other group consisted of non-registered residents of Shanghai. (5) Health insurance. There were two groups: One group had health insurance, and the other group did not have health insurance.

### Statistical analysis

**Descriptive analyses** were conducted to estimate the number of patients who reported health-related symptoms and healthcare-seeking behaviors per 1,000 residents in Shanghai. In addition, subgroup analyses were conducted by age, gender, economic status, census register and health insurance. Most health ecosystems are currently described on a monthly basis. To make the health ecosystem of Shanghai comparable with other places, this paper converted the disease prevalence and other indicators into monthly units according to the disease type of the respondents in Shanghai. Chronic diseases were considered both as those starting before the two-week period with symptoms continuing throughout this time frame and those with onset occurring during the considered two-week span and with symptoms lasting throughout this time frame. Furthermore, it was assumed that the annual hospitalization rate and annual surgery operation rate were evenly distributed in time within the year, and the indexes of hospitalization rate and surgery operation rate were converted into monthly units.

**Logistic regression analyses** were conducted to examine differences in reported health-related symptoms and healthcare-seeking behaviors by age, gender and area of residence. Outcome variables in this paper are including, (1) reported illnesses; (2) received treatment; (3) visited a physician in a medical institution (4) visited a physician in a primary medical institution; (5) took a self-healing approach; (6) received TCM treatment21-22; (7) had a hospital stay; or (8) had surgery. All outcome variables are dichotomous variables. Independent variables in multivariable analyses are including age, gender, economic status, census register, and health insurance. For each outcome variable, a logistic regression model logistic regression model was established to explain the influence of age, gender, income and other factors on each health outcome, and the difference of influence of various factors on different health outcomes was compared and analyzed. In addition, SAS 9.4 for Windows was used for data analysis in the study. The estimated numbers of those who experienced any health problem and/or used each kind of medical care per 1,000 people in two weeks was determined with a 95% confidence interval.

## Results

A total of 23,198 Shanghai residents participated in the Health Service Survey, 2018. According to the survey results, which considered the two week span prior to the survey, for every 1000 Shanghai residents, 422 people reported being sick, 411 received treatment, 274 went to medical institutions, 185 went to a primary medical institution, 82 took a self-healing approach, and 25 received TCM services. In the past year, of 1000 Shanghai residents, 82 people were hospitalized and 37 underwent surgery. Table 1 shows biweekly and yearly prevalence estimates for different forms of health services use stratified by age, gender, register and income to illustrate the corresponding effects of sociodemographics on care-utilization patterns. Table 2 shows the sample size for health-related symptom and healthcare seeking behaviors by demographic characteristics in Shanghai. Table 3 displays the adjusted odds ratios (ORs) of receiving care in each setting.

**Table 1:** Biweekly and yearly utilization estimates for the ecology of medical care in Shanghai in 2018 (persons per 1,000) by sociodemographic characteristics

Medical service	Biweekly utilization estimates						Yearly utilization estimates	
	Report illness	Take treatment	Visit a medical institution	Visit a primary medical institution	Self-healing	TCM	Hospital stay	Surgery
Overall	422 (415.9, 428.6)	411 (404.7, 417.4)	274 (268.4, 280)	185 (179.8, 189.8)	82 (78.6, 85.7)	25 (22.7, 27)	7 (6.6, 7.2)	3 (2.8, 3.2)
Age								
0–17	101 (88, 115.1)	98 (85.6, 112.4)	65 (54.9 77.3)	9 (5.4, 14.4)	38 (29.6, 46.9)	4 (1.4, 7.3)	2 (1.2, 2.3)	0.4 (0.2, 0.8)
18–39	104 (94.8, 113)	99 (90.2, 108.1)	64 (56.7, 71.4)	20 (15.9, 24.4)	36 (30.7, 41.9)	10 (7.1, 13.2)	4 (3.3, 4.4)	2.2 (1.8, 2.6)
40–64	415 (405.1, 425.7)	403 (393, 413.6)	257 (248.1, 266.4)	163 (155.7, 171.3)	85 (79.2, 91)	24 (20.7, 27.2)	6 (5.5, 6.0)	2.9 (2.6, 3.3)
65–79	669 (657.5, 680.7)	655 (642.7 666.2)	450 (438.1, 462.6)	333 (321.4, 344.7)	113 (104.9, 120.5)	38 (33.4, 42.9)	11 (9.9, 11.3)	4.4 (4.0, 4.9)
> = 80	742 (720.2, 763.6)	722 (699, 743.6)	**498**^**+**^ (473, 522.6)	379 (355.1, 403.3)	126 (109.8, 143)	44 (34.3, 55)	14 (12.5, 15.6)	2.9 (2.2, 3.7)
Sex								
Male	413 (404.2, 422.7)	402 (393, 411.4)	265 (257.2, 273.8)	178 (171, 185.4)	78 (73.5, 83.6)	18 (15.6, 20.6)	6 (6.0, 6.9)	2.7 (2.4, 3.0)
Female	430 (421.4, 439.1)	419 (410.2, 427.9)	282 (274.1, 290.2)	191 (183.8, 197.9)	85 (80.5, 90.5)	31 (27.8, 34.1)	7.2 (6.8, 7.7)	3.3 (3.0, 3.6)
Income								
< 100000	514 (504.7, 523.1)	**501**^**+**^ (491.8, 510.2)	336 (327.5, 344.9)	242 (233.8, 249.5)	98 (92.6, 103.6)	28 (25, 31.2)	8 (7.8, 8.7)	3.4 (3.1, 3.7)
100000–300000	344 (335, 353.3)	335 (325.7, 343.8)	222 (213.9, 229.9)	136 (129.3, 142.5)	70 (64.9, 74.7)	23 (19.7, 25.5)	5.7 (5.3, 6.1)	2.7 (2.4, 3.0)
300000–500000	251 (224.4, 279.6)	238 (211.7, 265.9)	150 (127.8, 173.4)	79 (63.2, 98)	42 (30.1, 56.2)	13 (7.1, 22.5)	,5 (3.5, 5.9)	2.1 (1.4, 3.1)
> 500000	211 (164.7, 262.5)	207 (161.5, 258.8)	140 (102.2, 186.2)	70 (43.4, 106.3)	39 (19.4, 68)	14 (3.8, 35.5)	5 (2.7, 7.5)	2.1 (0.8, 4.2)
Register								
In Shanghai	442 (435.4, 449.2)	431 (423.8, 437.5)	290 (283.3, 295.9)	199 (193, 204)	83 (79.5, 87.2)	26 (23.6, 28)	7 (6.7, 7.4)	3.0 (2.8, 3.2)
Out Shanghai	283 (266.4, 299.4)	275 (258.5, 291.2)	167 (153.6, 181)	90 (79.4, 100.4)	74 (64.5, 83.8)	18 (13.3, 23.3)	5.5 (4.8, 6.4)	2.9 (2.3, 3.5)
Insurance								
Yes	425 (418.6, 431.5)	414 (407.4, 420.3)	276 (270.2, 281.9)	187 (181.4, 191.6)	82 (78.8, 86)	25 (23, 27.1)	7 (6.6, 7.2)	3.0 (2.8, 3.2)
No	288 (248.3, 331.1)	278 (238.4, 320.3)	187 (152.9, 224.4)	104 (78, 134.5)	71 (49.3, 97.2)	10 (3.4, 24)	6 (4.1, 8.1)	2.6 (1.5, 4.2)

^+^*P* > 0.05

**Table 2:** Sample sizefor health-related symptom and healthcare seeking behaviors by demographic characteristics in Shanghai

	Report illness	Take treatment	Visit a medical institution	Visit a primary medical institution	Self-healing	TCM	Hospital stay	Surgery
**Age**								
**0–17** *n = 1971*	199	194	129	18	74	7	39	10
**18–39** *n = 4390*	455	434	280	87	158	43	202	114
**40–64** *n = 8838*	3671	3564	2273	1444	751	210	584	312
**65–79** *n = 6400*	4283	4189	2882	2131	720	243	813	340
**> = 80** *n = 1599*	1187	1154	796	606	201	70	269	55
**Sex**								
**Male** *n = 11032*	4561	4437	2928	1965	865	198	853	355
**Female** *n = 12166*	5234	5098	3432	2321	1039	375	1054	476
**Income**								
**< 100000** *n = 11437*	5877	5730	3845	2763	1121	320	1126	462
**100000–300000** *n = 10493*	3611	3512	2328	1425	731	236	711	337
** 300000–500000** *n = 983*	247	234	147	78	41	13	54	25
**> 500000** *n = 285*	60	59	40	20	11	4	16	7
**Register**								
**In Shanghai** *n = 20281*	8970	8734	5873	4025	1689	521	1713	731
**Out Shanghai** *n = 2917*	825	801	467	261	215	52	194	100
**Insurance**								
**Yes** *n = 22716*	9656	9401	6270	4236	1870	568	1873	816
**No** *n* = 482	139	134	90	50	34	5	34	15

**Table 3:** Adjusted odds ratio (95% CI) for health-related symptom and healthcare seeking behaviors by demographic characteristics in Shanghai

	Report illness	Take treatment	Visit a medical institution	Visit a primary medical institution	Self-healing	TCM	Hospital stay	Surgery
**Age**								
**0–17** *n = 1971*	1.00	1.00	1.00	1.00	1.00	1.00	1.00	1.00
**18–39** *n = 4390*	1.031 (0.865, 1.23)	1.005 (0.841, 1.202)	0.982 (0.791, 1.219)	0.448** (0.269, 0.747)	0.938 (0.708, 1.245)	2.335* (1.142, 4.772)	2.384*** (1.683, 3.376)	5.15*** (2.689, 9.858)
**40–64** *n = 8838*	5.923*** (5.078, 6.909)	5.787*** (4.953, 6.76)	4.65*** (3.86, 5.602)	0.052*** (0.033, 0.083)	2.283*** (1.784, 2.921)	5.917*** (3.03, 11.552)	3.43*** (2.468, 4.775)	7.139*** (3.787, 13.456)
**65–79** *n = 6400*	16.256*** (13.883, 19.035)	15.635*** (13.335, 18.333)	10.623*** (8.806, 12.815)	0.022*** (0.013, 0.034)	3.042*** (2.37, 3.905)	9.181*** (4.698, 17.941)	6.924*** (4.981, 9.624)	10.84*** (5.744, 20.457)
**> = 80** *n = 1599*	23.021*** (19.101, 27.746)	21.284*** (17.664, 25.645)	12.749*** (10.375, 15.667)	0.018*** (0.011, 0.029)	3.446*** (2.604, 4.559)	11.379*** (5.683, 22.782)	9.586*** (6.775, 13.563)	6.847*** (3.464, 13.536)
**Sex**								
**Male** *n = 11032*	1.00	1.00	1.00	1.00	1.00	1.00	1.00	1.00
**Female** *n = 12166*	1.01 (0.953, 1.072)	1.01 (0.955, 1.074)	1.04 (0.975, 1.103)	0.98 (0.909, 1.047)	1.07 (0.971, 1.174)	1.58 *** (1.344, 1.852)	1.08 (0.981, 1.188)	1.196* (1.039, 1.377)
**Income**								
**< 100000** *n = 11437*	1.00	1.00	1.00	1.00	1.00	1.00	1.00	1.00
**100000–300000** *n = 10493*	0.79 *** (0.742, 0.839)	0.79 *** (0.745, 0.843)	0.85 *** (0.793, 0.903)	1.31 *** (1.213, 1.409)	0.847** (0.766, 0.937)	1.01 (0.857, 1.188)	0.87* (0.782, 0.959)	0.94 (0.809, 1.088)
** 300000–500000** *n=983*	0.71 *** (0.598, 0.836)	0.68 *** (0.577, 0.809)	0.71 *** (0.584, 0.857)	1.65 *** (1.289, 2.119)	0.57 *** (0.412, 0.787)	0.64 (0.364, 1.123)	0.84 (0.628, 1.117)	0.85 (0.559, 1.281)
** >500000** *n=285*	0.51*** (0.371, 0.702)	0.53*** (0.382, 0.724)	0.64 * (0.445, 0.908)	1.95 ** (1.208, 3.146)	0.51 * (0.276, 0.935)	0.68 (0.248, 1.835)	0.83 (0.498, 1.396)	0.79 (0.369, 1.693)
**Register**								
**In Shanghai** *n = 20281*	1.00	1.00	1.00	1.00	1.00	1.00	1.00	1.00
**Out Shanghai** *n = 2917*	0.96 (0.867, 1.056)	0.96 (0.869, 1.059)	0.853** (0.764, 0.95)	1.346*** (1.169, 1.55)	1.227** (1.052, 1.431)	0.93 (0.702, 1.235)	1.09 (0.928, 1.28)	1.17 (0.938, 1.46)
**Insurance**								
**Yes** *n = 22716*	1.00	1.00	1.00	1.00	1.00	1.00	1.00	1.00
**No** *n* = 482	0.93 (0.739, 1.178)	0.92 (0.726, 1.161)	0.97 (0.755, 1.25)	1.14 (0.828, 1.574)	1.04 (0.728, 1.491)	0.70 (0.329, 1.50)	1.13 (0.787, 1.62)	1.13 (0.666, 1.909)

*Abbreviation*: *CI* Confidence interval

***P <0.001, **P <0.01, *P <0.05

Considering the disease type of the patients at the end of two weeks, patients with disease happened within the considered two-week period accounted for 8% of the total patients, patients with acute disease starting before the considered two-week period with symptoms continuing over the survey time frame accounted for 2%, and patients with chronic disease starting before the considered two-week period with symptoms continuing throughout survey time frame accounted for 90%.

Suppose that patients whose onset started two weeks ago and lasted for the last two weeks and whose chronic illness lasted for the last two weeks were still affected for a duration of one month. The incidence of new onset patients was stable over a period of time; that is, the monthly incidence was twice as high as the two-week incidence. Based on the above hypothesis, the number of cases in occurring in the studied two-week span was converted into the number of cases occurring in one month according to the type of patients. In addition, assuming that the hospitalization rate and operation rate are uniformly distributed in a year, the annual hospitalization rate and surgery rate can be converted into a monthly hospitalization rate and a monthly operation rate.

As a result, of 1000 individuals considered during a 1-month period, 444 reported being sick, 433 received treatment, 288 people went to medical institutions, 195 people went to primary medical institutions, 86 took a self-healing approach, 26 people received TCM services, 7 people were hospitalized, and 3 people underwent surgery.

## Discussion

Inspired by the publication of “The ecology of medical care” in 1961, many countries and regions have carried out health ecosystem research. However, there are few relevant studies on mainland China, with only one being published on the medical ecology of Beijing. In Beijing, on average per 1,000 adults, 295 had at least one symptom, 217 considered seeking medical care, 173 consulted a physician, 127 visited a hospital-based outpatient clinic, 43 visited a primary care physician, and 15 were hospitalized. Our paper considers Shanghai, another typical city in mainland China, as an example to describe the healthy ecosystem in China. There are two main differences in healthy ecosystems between Beijing 2012 and Shanghai 2018. First, Shanghai residents have utilized the medical system more, as evidenced by the higher number of reported cases and the higher number of visits. The possible reason for this difference may be the difference between the timing of the two reports (2012 vs. 2018). China’s medical field has developed rapidly in recent years. On the one hand, residents’ health awareness has been constantly improved [22], so increasing numbers of sick people have been reported. On the other hand, China’s medical and health system has been constantly improved, so people’s trust in the medical system has been constantly improved [23, 24]. Second, in the health ecosystem of Shanghai, the utilization of primary medical institutions is higher than that in Beijing, which is reflected in the fact that a larger proportion of patients chose to go to primary medical institutions for medical treatment. The reason for this difference may be that in recent years, China has strengthened the construction of grassroots medical institutions and established the family doctor system to guide patients at the grassroots to medical treatment. At present, some results have been achieved [25, 26].

Analyses of health ecologies of other countries have found that residents in different countries have different utilization structures of health ecology. We divided healthy ecosystems into three categories. The first category is the subjective health feedback of the population. The second category is the use of outpatient services. The third category is the utilization of inpatient services. The first type of subjective health feedback is closely related to local health awareness and the macro environment, so there is a huge difference in the number of reported cases per 1000 people in different countries. The South Korean health ecosystem (2012) [27] study estimated that 939 people reported cases per thousand, the Japanese health ecosystem (2013) [4] study estimated 794, the Taiwan health ecosystem (2005) [28] estimated 503, and the Hong Kong health ecosystem (2002) [29] estimated 567. In the second category, the utilization of out-patient services varied greatly. Different countries have different medical system designs, which lead to huge differences in the utilization structure of out-patient services. The third type of hospitalization service is not very different between countries because hospitalization is most common for complicated diseases, and doctors initiate hospitalization; thus, other phenomena will be greatly reduced, which can be reflected in the complicated disease structure in different regions. The utilization of inpatient services is basically the same in different countries because the probability of the occurrence of difficult diseases is relatively stable across the population.

Furthermore, the result of logistic regression analyses shows that age is a risk factor for disease and medical treatment. The older the age, the higher the risk of disease and the greater the possibility of medical resource utilization. Other national and local health ecology studies have reached similar conclusions [30]. Wealthy people were less likely to report illness and seek medical care in comparison with their lower income counterparts, and in other aspects of the medical resource utilization structure, there was no significant difference among people at different income levels. Studies in the Beijing area also found lower rates of reported illness among high-income groups. In addition, according to the Beijing study, compared with people with a monthly income of less than 1,000 yuan, people with a monthly income of more than 1,000 yuan and less than 5,000 yuan were more likely to make use of TCM. In Shanghai’s research, the outsiders were more likely to seek primary care and more likely to self-medicate, while these individuals were less likely to seek medical care at a medical facility. The main reason for this phenomenon is that the main social medical insurance in China has not yet realized the national pooling and unified settlement; thus, the non-local population living in Shanghai cannot directly enjoy the convenience of medical insurance in Shanghai, so their choice of medical treatment may be affected [31, 32]. In addition, the Shanghai medical service system implements a hierarchical pricing method: the basic medical institutions charge less than the county medical institutions, and the county medical institutions charge less than the city medical institutions. Therefore, driven by these two reasons, non-local patients in Shanghai are more likely to choose more economical primary medical institutions or self-treatment and are less likely to go to medical institutions for treatment. Currently, the Yangtze River Delta region, including Shanghai, is exploring the establishment of medical insurance integration to realize the homogenization of medical insurance among different regions [33], which is conducive to promoting the fair access to medical resources between non-local residents and local residents in Shanghai.

The limitation and future work of this paper are as followings. Firstly, the information regarding illness and medical treatment in this survey was primarily collected according to the time unit of a two-week period, while information on hospitalization and surgery was collected on a one-year basis. At present, most studies on health ecology are carried out by using one month as the research unit. In our study, data were collected for a two-week period, and values were estimated for a one-month period, which might have contributed some error in the estimates compared with relevant studies in other regions or countries. While data on hospitalization and surgery were collected for a year period, and values were estimated for a one-month period with the assumption that annual hospitalizations are evenly distributed over a year. Even through different diseases may have seasonal characteristics, this paper and the existing literature do not focus on the medical behavior in a specific season, but only on the overall medical treatment behavior in a month. Therefore, the simple assumption of average distribution is adopted. In the future work, the utilization of medical services by the population in different time periods within one year can be further analyzed and discussed.

Another limitation is that the survey adopted in this paper is retrospective, which inevitably have recall bias. However, the use of retrospective studies can reduce the cost of the survey considerably. To reduce the influence of recall error on the survey results, this survey adopted two weeks as the time unit, which can better reduce the deviation compared with using one month as the time unit. In addition, this article doesn’t analysis the evolution of the ecology of medical care in Shanghai in the timeline. The Health Service Survey of Shanghai has been conducted from six times, but it’s difficult to obtain original data of other recent rounds surveys, so this paper only analyzes and discusses the healthy ecology of Shanghai in 2018. In future research, we are going to including data from other recent rounds in the analysis to demonstrate the trend of health services utilization of Shanghai, which maybe can figure out other meaningful conclusions.

## Conclusions

**Fig. 1:**
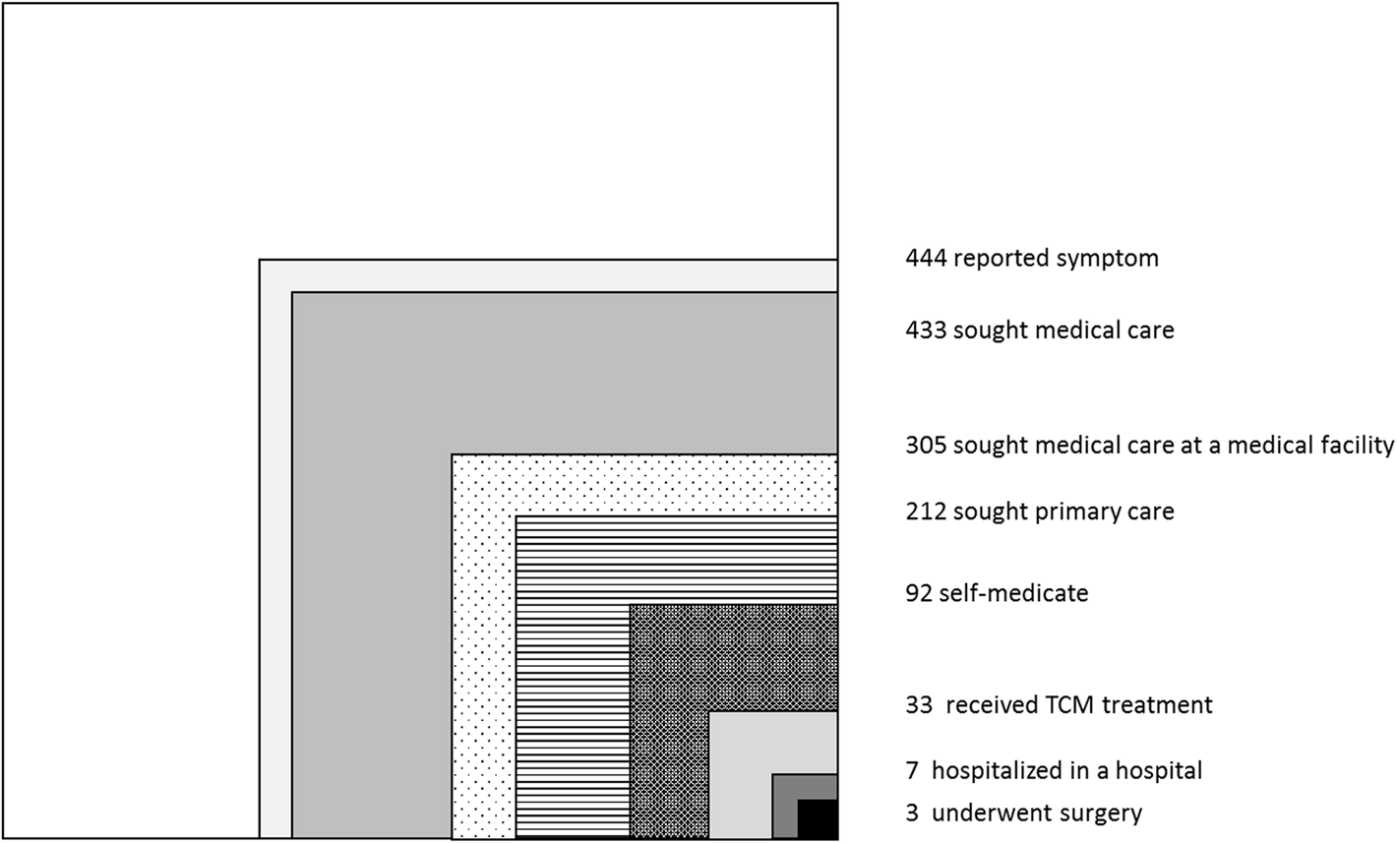
Monthly prevalence estimates of illness and the roles of different settings in the health-care u

To better understand the distribution and consumption patterns of resources in different ethnic groups and at different levels of economic development, this paper have mapped the ecology of healthcare in Shanghai in 2018 (Fig. 1).

As the population aged, the number of people reporting being sick and the number of people receiving medical services increased. According to the multivariable analysis results, age is a risk factor leading to population disease, medical treatment, self-medication, medical institution visits, TCM service, hospitalization and surgery. And it’s interesting to find that age is indeed a protective factor in the use of primary health care services, with the higher the grade, the lower the use of primary health care services. By gender, the number of cases and the number of people receiving medical services was similar. According to the results of multivariable analysis, no statistical significance was found in the effects of gender on disease, treatment, institutional visits, primary institutional visits, self-treatment, or hospitalization. However, women were statistically more likely to have TCM service and surgery than man. According to the income level statistics, as the income level increased, the number of patients and people receiving medical services showed a decreasing trend. According to the multivariable analysis, people earning more have a statistically significant reduction in the risk of getting sick and receiving medical treatment. However, people earning more are more likely to visit primary medical institutions. What’s more, no statistically significant was fond in the effect of income on TCM, hospital stay and surgery. In the comparison of the registered and non-registered residents of Shanghai, the number of reported patients and medical services received by the non-local population in Shanghai was lower than those of the local population. According to the results of multivariable analysis, compared with the local population in Shanghai, the probability of non-local people visiting medical institutions in Shanghai was lower and statistically significant. Further, the probability of non-local people going to primary medical institutions and utilizing self-healing approaches was higher, which was also statistically significant. Compared with the people who had health insurance, fewer uninsured people reported sickness and utilized healthcare services. According to the results of multivariable analysis, no statistical significance was found in the effects of insurance factors on population disease, treatment, institutional visits, primary medical institution visits, self-healing approaches, TCM services or hospitalization.

## Data Availability

The data will not be shared. All data used in this paper were supported by municipal governments of Shanghai, China. In order to o protect residents’ privacy, the municipal governments did not give us the authority to make the data public.

## Abbreviations

TCM: Traditional Chinese Medicine
ORs: Odds ratios
CI: Confidence interval
